# Multiple total hip arthroplasties in refractory immune thrombocytopenic purpura: A case report and literature review

**DOI:** 10.1097/MD.0000000000010308

**Published:** 2018-04-13

**Authors:** Yilun Tang, Yan Xu, Zhibin Shi, Xiaorong Ma, Lihong Fan, Kunzheng Wang, Xiaoqian Dang

**Affiliations:** a The FirstOrthopedics Department; b Department of Hematopathology, the Second Affiliated Hospital of Xi’an Jiaotong University, Xian, Shaanxi, China.

**Keywords:** fracture, hip joint, immune thrombocytopenic purpura, joint replacement, refractory

## Abstract

**Rationale:**

Refractory immune thrombocytopenic purpura (RITP) manifests as low platelet count, with a high risk of hemorrhage, treatment difficulty, and high mortality. Total hip arthroplasty (THA) in RITP is rarely reported. This study aimed to evaluate multiple THAs or revision total hip arthroplasties (RTHAs) in RITP.

**Patient concerns:**

The male patient with RITP was 54-year-old patient and hospitalized on September 21, 2009, with the main complaint of bilateral hip pain after traveling for 2 weeks. The patient had a history of ITP for 16-years, with no response to hormone therapy (after adequate hormone therapy, platelet count persistently remained below 30 × 10^9^/L). Two year prior to visit, the patient underwent splenectomy, and postoperative platelet persistently fluctuated around 10 to 20 × 10^9^/L. The patient did not undergo regular reexaminations, and declined immunosuppressants.

**Diagnoses:**

Femoral neck fracture; Refractory immune thrombocytopenic purpura (RITP).

**Interventions:**

A RITP patient with femoral neck fracture received 2 THAs and 1 RTHA. First THA indication was significant left dislocation of Garden III type. RTHA was performed following prosthetic loosening after left total hip arthroplasty. The second THA was prompted by non-healing of the old fracture, significant pain, and a low Harris score. Platelet count remained <20 × 10^9^/L, and conventional drugs, splenectomy, and platelet transfusion had no effects. A large gamma-globulin dose was administered preoperatively. When platelet increased to 75 × 10^9^/L, 2 THAs and 1 RTHA were successfully completed.

**Outcomes:**

Postsurgery, conventional management was applied; no severe complications occurred. The wound was well healed, with platelet count reduced to <15 × 10^9^/L at hospital discharge. The patient recovered, with a Harris score >80 at 1 year postsurgery.

**Lessons:**

Extremely low platelet count is a contraindication of surgery. In this patient, preoperative platelet count was <100 × 10^9^/L. Extended disease course and multiple operations lowered platelet count, and increased risk in surgery. However, high postoperative gamma-globulin dose impacted therapy, and all surgeries were successful, with no severe complications. The wound healed well, and the quality of life was significantly improved, demonstrating the feasibility and safety of this surgery. Multiple THA or RTHA surgeries are feasible and safe for RITP patients.

## 1 Introduction

Total hip arthroplasty (THA) is a commonly used treatment method for femoral head necrosis, coxitis, and femoral neck fracture; it greatly reduces disability and fatality rates, improving the quality of life. It is estimated that 572,000 THA surgeries will be performed every year till 2030 in USA. However, some internal medicine diseases are contraindications or relative contraindications of surgery, for example, various acute inflammatory diseases, hip with acute focus of infection, cardiopulmonary insufficiency, and blood coagulation disorders.

Immune thrombocytopenic purpura (ITP) is a common systemic disease mediated by immunity. Glucocorticoids are the first-line treatment drugs; therefore, the incidence of avascular necrosis of the femoral head is about 9% to 40%.^[1]^ Femoral neck fractures more easily occur; meanwhile, coxitis incidence gradually increases. Thus, the number of elderly individuals requiring THA would significantly increase. It has been reported that^[2,3]^ postoperative complications in ITP patients have high incidence rates, especially acute renal function failure, sepsis, hemorrhage, and pneumonia. About 30% of ITP patients have the refractory type (refractory immune thrombocytopenic purpura [RITP]), with no response to the traditional first-line treatments. RITP treatment is very challenging, with a 10-year fatality rate of 10% to 20%.^[4]^ In these patients, the surgical risk is very high, as well as complication and fatality rates. Inadequate treatment could cause massive bleeding and surgical complications. Meanwhile, safety during the perioperative period remains unclear. There are few reports of such patients receiving THA.^[5–8]^ Furthermore, nearly no report has described the same patient undergoing multiple THAs. The main aim of this study was to evaluate safety, feasibility, and efficacy of multiple THA or revision total hip arthroplasty (RTHA) in RITP patients.

A case of RITP with femoral neck fracture receiving 2 THAs and 1 RTHA was assessed. After multidisciplinary collaboration, the patient successfully recovered. The treatment process and related reports about RITP patients receiving multiple THAs are summarized below. The study protocol was approved by the Ethics Committees of the Second Affiliated Hospital of Xi’an Jiaotong University, Xian, and the participant provided written informed consent.

## 2 Case report

The male patient with RITP was born in Huxian, Shaanxi Province, of Han ethnicity. He was a farmer with no history of smoking or drinking. The surgery was coordinated by Prof. Xiaoqian Dang, a chief physician with extensive experience. All surgeries were performed by the same team.

The patient hospitalized 3 times had no anti-platelet antibodies, no autoantibodies and negative hepatitis test results; chromosome examination showed no significant abnormity. Thromboelastography showed low platelet function. Bone marrow examination showed active bone marrow hyperplasia, increased megakaryocytes, and decreased thrombocytopenic megakaryocytes, complying with the manifestations of thrombocytopenia. Previous glucocorticoid therapy had no efficacy in the patient. After splenectomy, postoperative platelet remained <20 × 10^9^/L for a long time. After consultation with the Hematopathology and Blood Transfusion Departments, and according to the diagnostic criteria of RITP in George et al^[9]^ and ITP International Working Group (IWG) in 2009,^[10]^ the patient was diagnosed with RITP.

Postoperative bleeding indices, such as drainage volume and wound bleeding condition, were assessed by the ITP-BAT score (ITP-specific bleeding assessment tool). Mucocutaneous and organ hemorrhages were observed, including nasal bleeding, gingival bleeding, oral mucosal bloody bulla, visceral bleeding (such as lung, gastrointestinal tract, and urogenital system), and central nervous system bleeding. Blood routine examination was performed every other day; when hemoglobin reduction did not reflect the dominant bleeding volume, or hemorrhagic symptoms occurred in corresponding organs, organ hemorrhage was highly suspicious.

**Figure 1:**
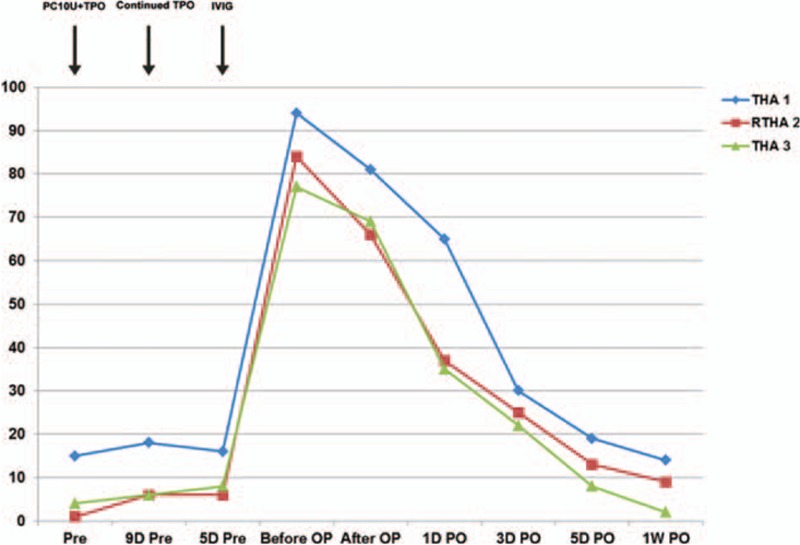
Platelet levels and therapeutic conditions in the perioperative period at different time points. Note: IVIG = gamma-globulin infusion, PC = platelet transfusion, TPO = recombinant human thrombopoietin, *Y*-axis = platelet count in 10^9^/L.

For the first left THA, a 54-year-old patient was hospitalized on September 21, 2009, with the main complaint of bilateral hip pain after traveling for 2 weeks. The patient had a history of ITP for 16-years, with no response to hormone therapy (after adequate hormone therapy, platelet count persistently remained below 30 × 10^9^/L). Two year prior to visit, the patient underwent splenectomy, and postoperative platelet persistently fluctuated around 10 to 20 × 10^9^/L. The patient did not undergo regular reexaminations, and declined immunosuppressants. The case was graded based on MSO (visible mucosae [M], skin [S] and organs [O]) description in disease history, referring to ITP-BAT13 by IWG in 2013 and hemorrhage scoring standards of World Health Organization.^[11]^ At this time, the patient was diagnosed with light hemorrhage (M = 1, S = 1, and O = 0), and had no history of hypertension, diabetes, coronary heart disease, or coagulation disorder. Physical examination revealed external rotation deformity in bilateral lower limbs, positive pressing bilateral groin pain, positive percussion pain in bilateral axial directions, shortened left lower limb by 0.5 cm, shortening of Bryant triangle bottom, top of the greater trochanter of femur higher than the Nelaton line, and limited bilateral hip joint motion. He rejected examination of motion due to pain. A visual analogue scale (VAS) pain score of 8 was obtained. Bilateral hip joint normotopia x-ray revealed bilateral femoral neck fracture, overt left dislocation, and no obvious right dislocation. The platelet level was 15 × 10^9^/L. The main diagnosis was bilateral femoral neck fracture: left, Garden III type; right, Garden I type. This diagnosis corresponded to RITP. Because left dislocation was obvious and of Garden III type, total hip replacement was selected. The right side was of Garden I type; platelet count was severely reduced, and the risk of synchronous bilateral THA was too high. After discussion with family members, conservative treatment for the right side was selected, and surgery was decided according to the recovery condition. Therefore, the therapeutic option selected for the first bilateral femoral neck fracture was THA on the left side under general anesthesia. He was administered continuous irradiated platelets by transfusion (10 U daily for 3 days) and recombinant human thrombopoietin (rhTPO, 15,000 U, once daily, subcutaneous injection for 7 days). However, this therapy was not successful. Then, a high gamma-globulin (IVIG) dose was intravenously injected (2 g/kg, 400 mg/[kg d] × 5 d). The patient's body weight was 70 kg, for a dose of 27.5 g/d × 5 days. No infusion reactions, such as pyrexia, shiver, and rash, occurred. The highest platelet level before the first THA was 94 × 10^9^/L. Preoperative maximum values were obtained at 8:00 am on the surgery day; surgery time was around 9:00 am (Fig. 1, Table 1). In the perioperative period, hemostatic and gastric acid inhibitors were used. Besides, from 1 hour before surgery to 3 days postoperation, antibiotics were used to prevent infection.

**Table 1:**
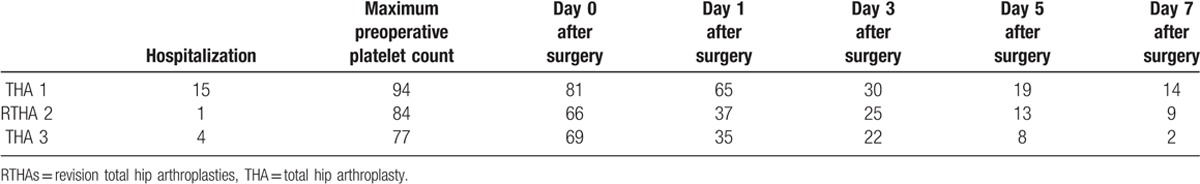
Platelet counts during the perioperative period at different time points (×10^9^/L).

Total hip prosthesis was selected during the surgery (AESCULAP, biological artificial hip prosthesis, No. 11 femoral prosthesis stem, No. 54 acetabular prosthesis, and No. 28 femoral head). Conservative treatment was selected for the right side. During the operation, the bleeding blood vessels were ligated as much as possible. Electric coagulation hemostasis was used with caution to prevent electric clots from falling off and causing postoperative bleeding. Surgery duration was 48 minutes, for an intraoperative blood volume of 200 mL; volume of drainage was 300 mL, and no blood transfusion was performed (Table 2). Postoperative platelet count was 81 × 10^9^/L. Twelve hours after surgery, enoxaparin sodium at 4000 IU/d was administered as anticoagulant therapy. An intermittent pneumatic compression device was used to physically prevent thrombosis. Enoxaparin sodium was discontinued when platelet count decreased to 14 × 10^9^/L at 7 days postsurgery. Symptomatic treatments, including fluid replacement, pain relief, and anti-infection, were provided to the patient. Time to stitch removal was 12 days, and the length of hospital stay was 24 days. Postoperative symptoms were greatly improved, and the patient recovered well without significant complications. Anterioposterior and lateral x-ray scans after conventional surgery were obtained, and the patient was reexamined at 6 weeks, 12 weeks, 6 months, and 1 year postsurgery, respectively. The patient was encouraged to perform passive motor function training on the affected limb at 2 days postsurgery. In the early stage, the patient practiced quadriceps exercises, as well as hip and knee flexion activities. After 2 to 3 weeks, the patient could stand with a stick without weight-bearing on the affected limb. After 3 months, he could practice walking with stick, and dropped the stick at 6 months postoperation, walking with weight-bearing on the affected limb. The VAS pain score at 1 year after surgery was 3, for a Harris score of 85. During follow-up, no complications, such as prosthesis loosening, fracture, infection around the prosthesis, occurred, and no venous thrombosis or bleeding was detected. The patient was not reexamined after 1 year.

**Table 2:**
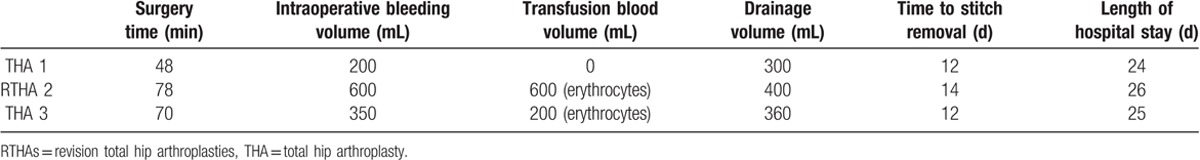
Intraoperative, bleeding, and wound conditions in the 3 surgeries.

**Figure 2:**
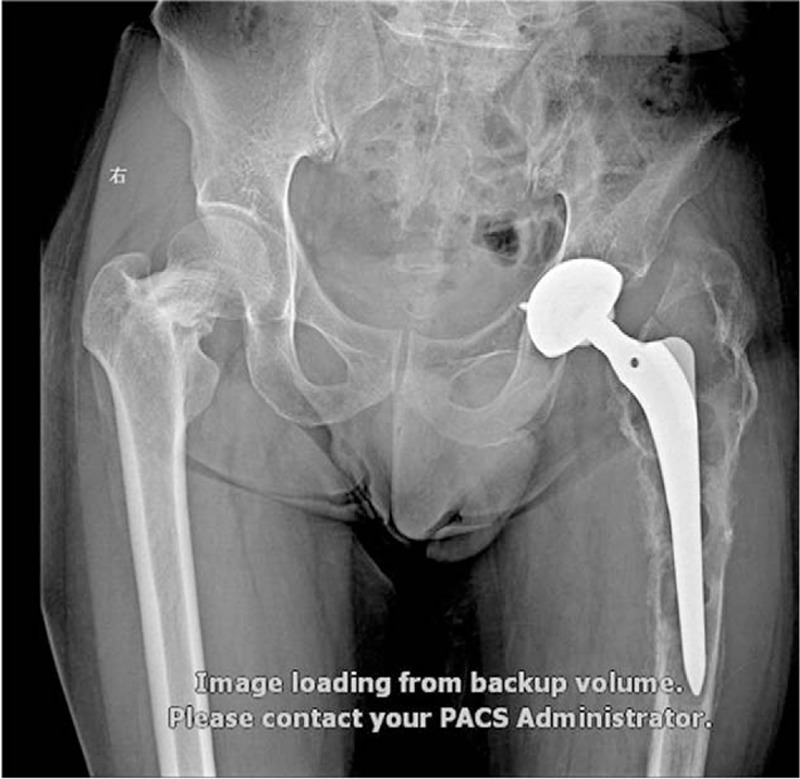
X-ray image before the second RTHA on the left side and before the third THA on the right side. RTHA = revision total hip arthroplasty, THA = total hip arthroplasty.

**Figure 3:**
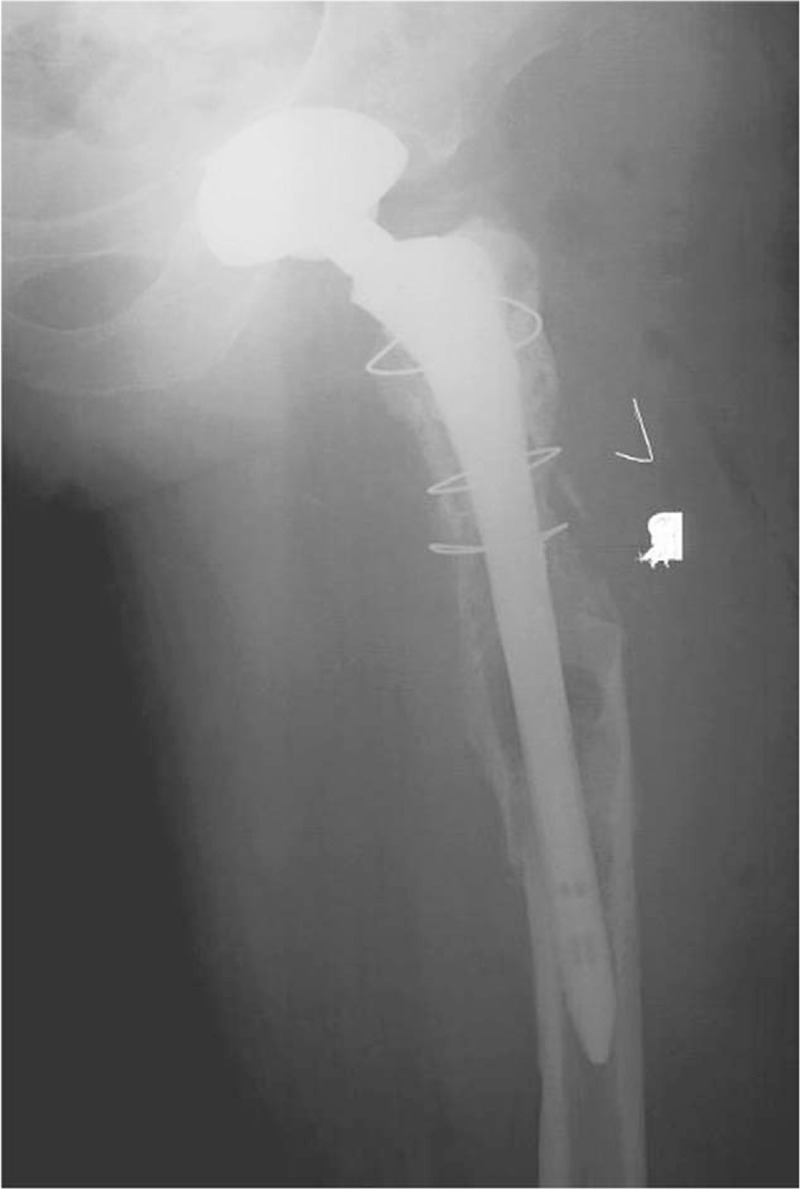
X-ray image after the second RTHA on the left side. RTHA = revision total hip arthroplasty.

For the second left RTHA, the patient was hospitalized on February 25, 2014, at the age of 59, with the main complaint of left hip pain for 2 years. Before surgery, according to ITP-BAT scoring, the patient had moderate bleeding (M = 2, S = 1, and O = 0). Physical examination revealed limited left lower limb activity, with a range of motion between 0° and 60°. Sensation of left lower limb skin was good, with a muscle strength of IV; the VAS pain score was 5. Bilateral hip joint normotopia and left thighbone anteroposterior and lateral x-ray scans revealed left hip joint alteration after replacement; bone absorption, cortex thinning, periosteal proliferation, and medullary cavity enlargement occurred in left upper femur, which might be reactive thighbone adsorption on prosthesis. Right femoral neck showed unnatural cortical deformation, which might reflect an old fracture (Fig. 2). Platelet count before hospitalization was 1 × 10^9^/L. The main diagnosis was prosthesis loosening after left total hip arthroplasty, with an old fracture in right femoral neck, indicating refractory ITP. He was administered continuous irradiated platelets by transfusion (10 U daily for 3 days) and recombinant human thrombopoietin (rhTPO, 15,000 U, once daily, subcutaneous injection for 7 days). However, this therapy was not successful. Then, a high gamma-globulin (IVIG) dose was intravenously injected (2 g/kg). The patient's body weight was 70 kg, for a dose of 27.5 g/d × 5 days. The highest platelet level before the first THA was 84 × 10^9^/L (Fig. 1, Table 1). In the perioperative period, hemostatic and gastric acid inhibitors were used. Besides, from 1 hour before surgery to 3 days postoperation, antibiotics were used to prevent infection. The patient underwent RTHA on the left side under general anesthesia, and a total hip joint prosthesis system was used during surgery (AESCULAP, cement type prosthesis, No. 13 long-stem, No. 28 femoral head, and No. 50 joint of bone). Surgery time was 78 minutes, with an intraoperative blood volume of 600 mL; blood transfusion and drainage volumes were 600 and 400 mL, respectively (Table 2). Postoperative platelet count was 66 × 10^9^/L. Twelve hours after surgery, enoxaparin sodium at 4000 IU/d was provided as anticoagulant therapy. An intermittent pneumatic compression device was used to physically prevent thrombosis. Enoxaparin sodium was discontinued when platelet count decreased to 9 × 10^9^/L at 7 days after surgery. Symptomatic treatments, including fluid replacement, pain relief, and anti-infection, were provided to the patient. Time to stitch removal was 14 days, and the length of hospital stay was 26 days. The postoperative symptoms were greatly improved, and the patient recovered well without significant complications (Fig. 3). The VAS score at 1 year postsurgery was 2, for a Harris score of 80. During the follow-up period, no complications occurred, including prosthesis loosening, fracture, and infection around the prosthesis, and no venous thrombosis or bleeding was found.

**Figure 4:**
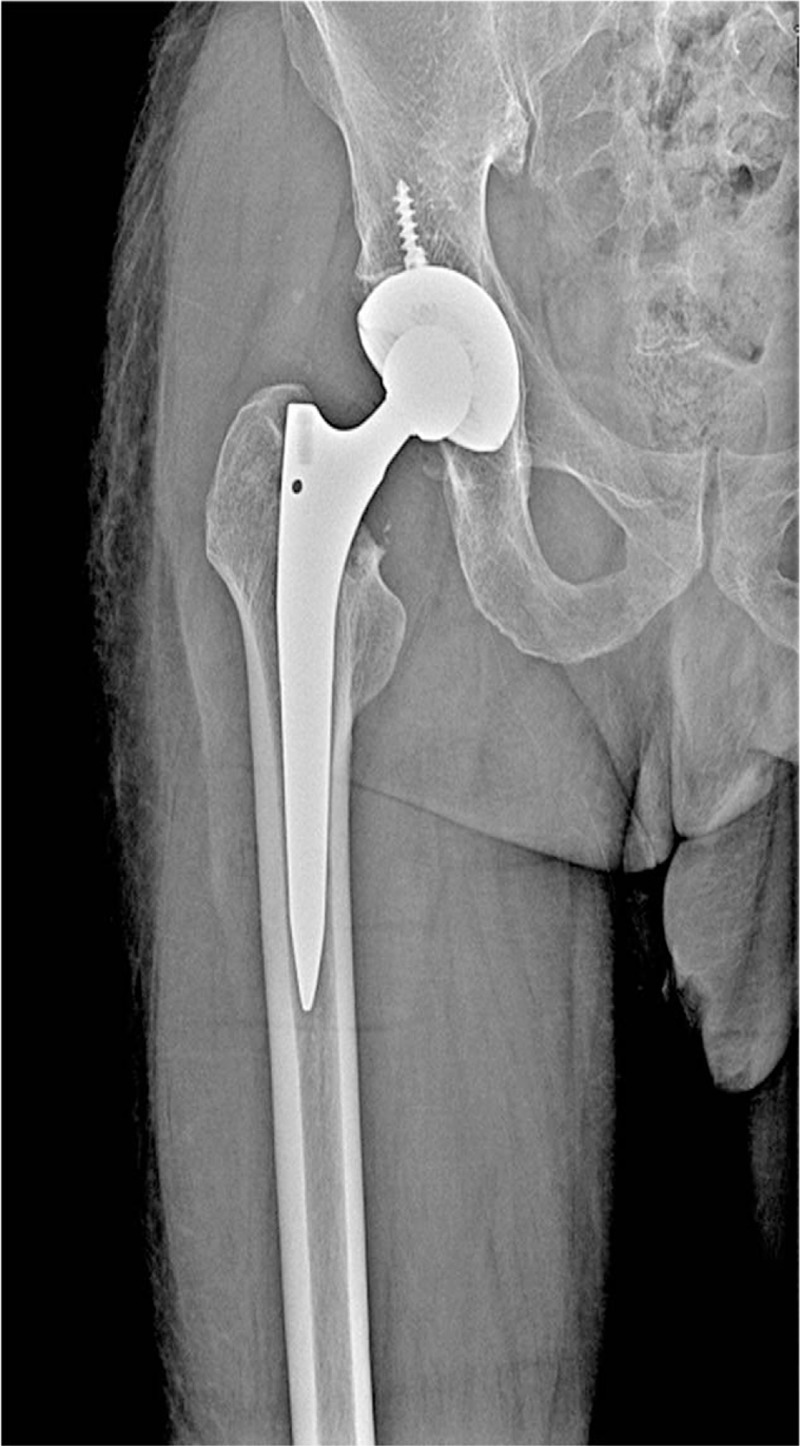
X-ray image after the third THA on the right side. THA = total hip arthroplasty.

For the third right THA, the patient was hospitalized on October 12, 2016 at the age of 61-year, with the chief complain of pain in right hip for 1 month. Before surgery, according to ITP-BAT scoring, the patient had moderate bleeding (M = 2, S = 1, and O = 0). Seven years ago, the patient underwent total hip replacement on the left side because of bilateral femoral neck fracture, and RTHA on the left side 2 years ago. Physical examination revealed no obvious hemorrhagic spots or ecchymosis in systemic skin mucosa; there was no deformity in right lower limb appearance. Pressing pain in right midpoint of the groin and longitudinal percussion pain were positive, with limited right hip joint motion. The patient rejected examination due to pain; the VAS pain score was 6. Platelet count before hospitalization was 4 × 10^9^/L. X-ray scan showed an old right femoral neck fracture, and alterations after THA on the left side (Fig. 2). The main diagnosis was old fracture in right femoral neck after left THA, reflecting refractory ITP. Surgical indications were non-healing of an old fracture in right femoral neck, significant pain, and low Harris score (score = 30). Therefore, joint replacement was selected. Given the relatively young age, HTA on the right side under general anesthesia was performed. As the same as the previous 2 operations, he was administered continuous irradiated platelets by transfusion (10 U daily for 3 days) and recombinant human thrombopoietin (rhTPO, 15,000 U/d, subcutaneous injection for 7 days). However, this therapy was not successful. Then, a high gamma-globulin (IVIG) dose was intravenously injected (2 g/kg). The patient's body weight was 70 kg, for a dose of 27.5 g/d × 5 days. The highest platelet level before the first THA was 77 × 10^9^/L (Fig. 1, Table 1). In the perioperative period, hemostatic and gastric acid inhibitors were used. Besides, from 1 hour before surgery to 3 days postoperation, antibiotics were used to prevent infection. A total hip prosthesis system (joint, biological artificial hip prosthesis, No. 3 femoral prosthesis stem, No. 54 acetabular prosthesis, and No. 28 femoral head) was used. Surgery time was 70 minutes, for an intraoperative blood volume of 350 mL; blood transfusion and drainage volumes were 200 and 360 mL, respectively (Table 2). Postoperative platelet count was 69 × 10^9^/L. Twelve hours after surgery, enoxaparin sodium at 4000 IU/d was administered as anticoagulant therapy. An intermittent pneumatic compression device was used to physically prevent thrombosis. Enoxaparin sodium was discontinued when platelet count decreased to 2 × 10^9^/L at 7 days after surgery. Symptomatic treatments, including fluid replacement, pain relief, and anti-infection, were provided to the patient. Time to stitch removal was 12 days, and the length of hospital stay was 25 days. The postoperative symptoms were greatly improved, and the patient recovered well without significant complications (Fig. 4). Postoperative 1-year VAS score was 2, for a Harris score of 90; there was no adverse event during follow-up.

## 3 Discussion

In clinic, severe low platelet count is a contraindication of surgery; indeed, the surgical difficulty and risk are very high in this case. When a patient with severe thrombocytopenia needs THA, the orthopedist often faces conflicting choices, given the possibility of severe postoperative hemorrhage. Thus, many patients wave the surgical opportunity. The commonly used therapy in such cases is preoperative conservative treatment, including adrenocortical hormone, immunosuppressant, danazol, immunoglobulin, and splenectomy applications, ensuring that platelet levels can reach a relatively safe count. However, this extends the length of hospital stay, or even increases the incidence rates of complications. When surgery is necessary, according to preoperative platelet count, the adrenocortical hormone dose, scope of surgery, and surgical method, as well as preoperative preparation, intraoperative process, and precautions for postoperative complications should be optimized. We reported and discussed the feasibility and perioperative safety of multiple THAs or RTHA in a patient with RITP.

The pathogenesis of RITP does not only correlate with humoral immunity, but also has a certain relationship with cellular immunity and megakaryocyte abnormity. George et al^[9]^ proposed the following diagnostic criteria for RITP: no response to glucocorticoids and splenectomy, age >10 years old, disease course >3 months, no other diseases causing thrombocytopenia, and platelet count <50 × 10^9^/L. In 2009, the ITP IWG designed 3 standards for RITP diagnosis^[10]^: invalid or relapsed ITP patients after splenectomy; ITP patients requiring small amounts of drugs such as glucocorticoids to reduce hemorrhagic risk; ITP patients with the exception of other reasons for thrombocytopenia. The ITP Consensus of Chinese Experts (2012) pointed out that the following factors could increase hemorrhagic risk: old age and prolonged disease course; platelet dysfunction; coagulation factor deficiency; uncontrolled hypertension; surgical operation or trauma; infection; required administration of anticoagulation drugs such as aspirin, non-steroid anti-inflammatory drugs, and warfarin. RITP patients often show delayed healing or chronicity, and platelet count remains low for a long time. Besides, the survival time of platelets is shortened, and hemorrhage easily occurs. For example, severe visceral or intracranial hemorrhage may be life threatening. About 5% of patients may die from intracranial hemorrhage, and the incidence of severe hemorrhage in elderly individuals is even higher. The disease course of chronic RITP patients is long, and poor treatment efficacy is obtained. Interestingly, patient prognosis is associated with platelet count.^[12,13]^ Meanwhile, hemorrhagic risk is positively correlated with the severity of thrombocytopenia, with highest risk for platelet counts <20 × 10^9^/L. The platelet count is lower preoperative, the risk of blood loss will be higher intraoperative, and the operation will be more difficult. The current patient was male, with platelet levels remaining below 20 × 10^9^/L for a long time. The therapeutic effect was poor, and he received THA (or RTHA) 3 times; therefore, hemorrhagic risk was very high, gradually increasing with age and disease course.

Generally, when platelet count is >80 × 10^9^/L, the risk of abnormal hemorrhage is very low. Meanwhile, with platelet count <50 × 10^9^/L, excessive bleeding or hematoma formation easily occurs during or after surgery. When platelet count is <25 × 10^9^/L, hemorrhage occurs spontaneously and even the operation cannot be performed. Shih et al also demonstrated that decreased platelet count is a risk factor for complications after arthroplasty.^[14–16]^ Low platelet count could lead to delayed or no healing, subcutaneous hematoma, and soft tissue and muscle hematoma, which would further cause secondary wound infection and unsuccessful surgery. Acute massive blood loss both during and after surgery would cause hemorrhagic shock, further threatening the patients’ lives. Due to extreme low platelet count before surgery, the platelets transfused preoperatively might be rapidly destroyed in vivo. Gauze should be continuously used for intraoperative incision and wound, which leads to extended surgery time. On the other hand, due to extremely low platelet count, treatment efficacy is poor. Thus, patients have to use hormones for a long time, which may cause side effects, for example, obesity. Meanwhile, excessive hormone administration may cause fragility and bleeding in tissues. In addition, it is more difficult to expose, separate, and ligate the vessels, leading to longer surgery time and higher hemorrhagic risk. Therefore, when platelet count is <50 × 10^9^/L in femoral neck fracture patients with RITP, it must be increased to hemostatic levels at 24 hours presurgery (70 × 10^9^/L). During surgery and within 72 hours thereof, platelet levels must remain above 50 × 10^9^/L. RITP treatment is indeed very difficult. The current treatment does not yield good results, and obvious side effects are possible. In patients with RITP, immunoglobulins, cytokines, interferons, and monoclonal antibodies are the commonly used therapeutics with good efficacy. The commonly used preoperative treatment modalities in RITP patients include: gamma-globulins; transfusion of fresh platelets; transfusion of whole blood; immunosuppressants (e.g., cyclosporine A, azathioprine, and cyclophosphamide); danazol; thrombopoietin (TPO); monoclonal antibodies (e.g., Rituximab, anti-CD52 monoclonal antibody, Daclizumab, and anti-CD40L monoclonal antibody). Blood or platelet transfusion can directly increase platelet count and the amounts of blood coagulation factors, but could spread blood-borne diseases. Immunosuppressants inhibit the immune system, increasing the number of patients with the complication of malignant diseases by 5 to 10 times. Danazol has a high effective rate in female patients, and lasts from weeks to months. However, there are many related side effects, such as headache, liver function damage, and voice change. Monoclonal antibodies are very costly, with multiple side effects; indeed, some are still under clinical trials, with unclear efficacy.

The current patient received a high dose of immunoglobulins by intravenous injection (HD-IVIg) before all 3 surgeries. Mayer et al^[17]^ demonstrated that high gamma-globulin dose (1–2 g/kg) is significantly effective in rapidly increasing platelet concentrations. Referring to the Consensus of Chinese Experts on diagnosis and treatment of adult primary immune thromboaytopenia,^[18]^ high gamma-globulin dose administration is recommended in China (2 g/kg, 400 mg/[kg d] × 5d). The current study indicated that at 5 to 10 days after intravenous injection of gamma-globulins in the ITP patient, platelet levels were significantly increased, and remained high for 10 days. Indeed, such treatment is considered to be effective in rapidly increasing platelet count. HD-IVIg could achieve a temporary increase of platelet count by binding the Fc receptor of phagocytes or antiplatelet antibodies, which are commonly used in the preparation of major surgeries (e.g., cardiac surgery). However, HD-IVIg could increase platelet activity, likely enhancing the risk of thrombosis, especially in the elderly. Patients administered gamma-globulins before surgery easily develop systemic sepsis; therefore, sterile conditions should be paid attention to. In case of significantly reduced platelet count, intramuscular injection should be avoided for preoperative administration.

Suzuki et al^[19]^ reported a case of ITP accompanied by steroid-induced avascular necrosis of the femoral head who underwent THA; preoperative platelet count was 25,000/μL. After administration of high gamma-globulin dose and platelet transfusion, preoperative platelet levels reached 94,000/μL. In the latter patient, the operation was successful, and no complications occurred in the perioperative period; at hospital discharge, platelet count was reduced to 34,000/μL. Recovery was good in the 3-year follow-up period. The patient could walk, and the implant showed no loosening. Nezu et al^[5]^ reported that first- and second-line drugs have no efficacy while evaluating a 42-year-old RITP patient. After administration of combined vincristine and colchicine, platelet levels were significantly increased, and successful hip replacement was performed. Kim et al^[6]^ successfully completed THA for ITP-related femoral head necrosis. Singhal et al^[7]^ reported a 61-year-old RITP patient that successfully underwent TKA. Lim et al^[8]^ compared 11 ITP (15 THA cases) and 22 non-ITP (30 THA cases) patients in a retrospective study. They found no significant differences in surgery time, intraoperative bleeding, drainage volume, length of hospital stay, and rehospitalization rate between the 2 groups. Besides, adverse events did not increase. All the ITP patients reported in the literature only underwent THA once, with most of them (11/19) having preoperative platelet levels higher than 100 × 10^9^/L after treatment. In this study, the patient received 3 surgeries with strict surgical indications. Preoperative platelet levels were never higher than 100 × 10^9^/L at any operation. Moreover, with extended disease course and increased number of operations, platelet amounts became lower, increasing the surgical risk. Platelet count in the RITP patient presented here remained below 20 × 10^9^/L for a long time. Furthermore, platelet count did not increase at 3 days after continuous transfusion of irradiated platelets, indicating failed therapy. Thus, multiple platelet transfusions might induce antiplatelet antibody production. After treatment with a high gamma-globulin dose, preoperative platelet count reached 75 × 10^9^/L, and the surgery was successful. In the perioperative period, hemostatic therapy was enforced. Postoperative platelet count was significantly decreased to 65, 37, and 35 × 10^9^/L within 1 day after the 3 surgeries, respectively, and to 19, 13, and 8 × 10^9^/L at 5 days after the operations, respectively. Drainage volumes were 300 to 400 mL, and no severe bleeding occurred. The wound healed well, and platelet count was reduced to <15 × 10^9^/L at hospital discharge. After surgery, the patient recovered well, and no bleeding or thrombus occurred. The Harris score at 1 year was >80; the pain index was significantly improved, without affecting daily activities. The quality of life was significantly improved as well in this patient, suggesting that the intervention was effective, feasible, and safe.

The current patient receiving hip joint replacement was evaluated according to Caprini thrombotic risk factors, and a score >5 was obtained. Thus, this case belonged to the extremely high risk group. The risk of deep vein thrombosis (DVT) after surgery is about 40% to 80%, with a fatality rate of about 1% to 5%. Besides, the current RITP patient had undergone splenectomy, therefore increasing the risk of phlebothrombosis.^[20]^ In this patient, medicinal prevention and physical intervention were implemented after surgery. The patient received enoxaparin sodium at 12 hours after surgery for the 3 surgeries by subcutaneous injection (4000 IU, once daily). Because platelet count at 5 days postsurgery decreases to <20 × 10^9^/L in all 3 surgeries, anticoagulation therapy was administered for 1 week. The patient was encouraged to perform passive motor function training on the affected limb at 2 days after surgery. No significant hemorrhagic complications occurred during follow-up, and no DVT was formed.

Overall, a RITP patient successfully underwent 2 THAs and 1 RTHA, and bleeding score and severity gradually increased, as well as surgical risk and difficulty. Postoperative drainage and bleeding volumes increased after 2 THA surgeries compared with previous values; meanwhile, RTHA caused greater trauma, with higher surgical difficulty, more bleeding, and elevated drainage volumes compared with THA. However, no severe complications occurred. Considering adequate preoperative preparation, surgical operation, and postoperative management, the feasibility and safety of THA in refractory ITP was demonstrated. Such effectiveness greatly improved the patient's quality of life.

## Acknowledgments

The investigations were conducted at the Second Affiliated Hospital of Xi’an Jiao Tong University, China.

## Author contributions

**Conceptualization:** Xiaoqian Dang.

**Investigation:** Yilun Tang, Yan Xu, Zhibin Shi, Xiaorong Ma, Lihong Fan, Kunzheng Wang.

**Methodology:** Yilun Tang, Yan Xu, Zhibin Shi, Xiaorong Ma, Lihong Fan, Kunzheng Wang.

**Writing – original draft:** Yilun Tang, Yan Xu.

**Writing – review & editing:** Yilun Tang, Yan Xu.
